# Association of endometriosis with genital human papillomavirus infection in US women: a national population-based study

**DOI:** 10.1038/s41598-023-35153-0

**Published:** 2023-05-17

**Authors:** Yun Soo Hong, Jihwan Park, Hoon Kim

**Affiliations:** 1 Departments of Epidemiology and Medicine and Welch Center for Prevention, Epidemiology and Clinical Research, Johns Hopkins University Bloomberg School of Public Health, 2024 E. Monument St., Baltimore, MD 21205 USA; 2 Department of Obstetrics and Gynecology, Seoul National University College of Medicine, 101 Daehak-ro, Jongno-gu, Seoul, 03080 Korea; 3 Department of Obstetrics and Gynecology, Seoul National University Hospital, 101 Daehak-ro, Jongno-gu, Seoul, 03080 Korea

**Keywords:** Diseases, Medical research

## Abstract

The prevalence of genital human papillomavirus (HPV) in women with endometriosis has never been reported in a national representative survey. We aimed to investigate the association of endometriosis with the prevalence of HPV. We analyzed the data on 1768 women (representing 43,824,157 women) in the United States aged 20–54 years from the National Health and Nutrition Examination Survey in the prevaccination era (2003–2006). The diagnosis of endometriosis was based on a self-report. The prevalence of any HPV in women with endometriosis did not differ from that in women without endometriosis after controlling for potential confounders such as age, ethnicity, family income, marital status, and the number of deliveries (adjusted prevalence ratio (aPR) 0.84, 95% confidence interval (CI) 0.61–1.15). No significant association was found between the prevalence of high-risk HPV and the diagnosis of endometriosis (aPR 0.71, 95% CI 0.44–1.14). If the participants were not covered by health insurance, the prevalence of any HPV infection in women with endometriosis was higher than in those without endometriosis (aPR 1.44, 95% CI 0.94–2.20). In contrast, in a subgroup who had health insurance, a lower prevalence of any HPV infection was observed in women with endometriosis (aPR 0.71, 95% CI 0.50–1.03), and P for interaction was statistically significant (P = 0.01). There was no association between endometriosis and HPV infection in this study of HPV vaccine-naïve women of reproductive age. The association was not different by the type of HPV. However, access to healthcare may modify the association between endometriosis and HPV infection.

## Introduction

Endometriosis, the presence of ectopic endometrial tissue outside the uterine cavity, is a relatively common gynecologic disorder affecting approximately 10% of reproductive-age women^1^. Although it is generally considered a benign disease associated with symptoms including infertility, dysmenorrhea, and chronic pelvic pain^2^, endometriosis shares common features with malignant tumors, such as neovascularization, invasion to normal tissue, and decreased apoptosis^3,4^.

Cervical cancer is one of the most common gynecologic cancers in the US. Although its incidence rate has decreased significantly from 12.3 cases to 6.5 cases per 100,000 women over the last 40 years^5^, it is estimated that in 2021 there would be over 14,000 cases of newly diagnosed invasive cervical cancer, and approximately 4300 women would die from this cancer in the US^6^. In recent meta-analyses, the relative risk of cervical cancer in women with endometriosis was 0.67 to 0.78 compared to those without endometriosis^7–9^. In several retrospective cohort studies, also, women with endometriosis had a lower standardized incidence ratio of cervical cancer^10–12^. However, the biological mechanism of cervical cancer development in women with endometriosis has not been well investigated.

Human papillomavirus (HPV) is the single most important cause of cervical cancer^13^, causing about 90% of all cervical cancers^14^. The risk of HPV infection may be lower in endometriosis due to decreased sexual activity^15^. However, at the same time, the risk may be higher due to changes in the immune reaction in endometriosis^16^. Furthermore, several studies reported abnormal natural killer cell activity^17,18^, T helper 1/T helper 2 equilibrium shift^19,20^, or translocation of T regulatory cells^21^ in endometriosis. Therefore, endometriosis may be associated with HPV infection, but the prevalence of HPV infection in women with endometriosis has been reported only in small studies and the findings were not consistent^22–24^. Therefore, it is important to better understand the natural association of endometriosis with HPV prevalence in a large population before the introduction of HPV vaccination.

We, thus, aimed to evaluate the association between endometriosis and the prevalence of HPV infection in a nationally representative, cross-sectional National Health and Nutrition Examination Survey (NHANES) between 2003 and 2006 before the approval of HPV vaccination by the US Food and Drug Administration (FDA).

## Results

Of 1768 females included in the study, 129 women reported physician-diagnosed endometriosis, representing 9.5% (95% CI 7.3–12.3%) of females aged 20–54 years in the general US population (Table 1 and Table S1). Women with endometriosis were significantly more likely to be older, non-Hispanic Whites, and current smokers compared to those without endometriosis. The lifetime number of male sex partners and age at first sexual intercourse were not different by the diagnosis of endometriosis. The frequency of vaginal or anal sex in the past 12 months, however, was significantly lower in women with endometriosis compared to those without endometriosis (P < 0.001).

**Table 1:** Baseline characteristics of study participants.

Variables	All	Women without endometriosis	Women with endometriosis	P value
No. of participants	1768	1639	129	
No. of participants, weighted	43,824,157	39,644,285	4,179,872	
HPV (+)	822 (42.6%)	765 (43.2%)	57 (36.9%)	0.35
High risk HPV (+)	520 (26.7%)	489 (27.4%)	31 (19.8%)	0.15
Age categories, weighted %				
20–34	717 (30.0%)	697 (31.7%)	20 (13.3%)	< 0.001
35–54	1051 (70.0%)	942 (68.3%)	109 (86.7%)	
Race/ethnicity, weighted %				
Hispanic	464 (12.9%)	452 (13.7%)	12 (4.9%)	< 0.001
Non-Hispanic White	775 (67.5%)	692 (66.0%)	83 (81.7%)	
Non-Hispanic Black	459 (14.6%)	428 (15.1%)	31 (10.2%)	
Other races	70 (5.1%)	67 (5.3%)	3 (3.3%)	
Education, weighted %				
Less than high school	434 (15.8%)	417 (16.5%)	17 (9.6%)	0.20
High school graduate	423 (24.8%)	391 (24.1%)	32 (31.5%)	
Some college or AA degree	603 (37.5%)	552 (37.5%)	51 (36.8%)	
College graduate or above	308 (21.9%)	279 (21.9%)	29 (22.0%)	
Ratio of family income to poverty, weighted %				
< 1.5	674 (26.3%)	637 (26.4%)	37 (25.7%)	0.48
1.5 ≤ < 3.0	437 (25.0%)	411 (25.5%)	26 (20.5%)	
≥ 3.0	657 (48.7%)	591 (48.1%)	66 (53.7%)	
Marital status, weighted %				
Married/living with partners	1210 (72.3%)	1129 (73.0%)	81 (65.9%)	0.09
Widowed/divorced/separated	313 (18.1%)	277 (17.3%)	36 (26.2%)	
Never married	245 (9.5%)	233 (9.7%)	12 (7.9%)	
Number of vaginal/cesarean deliveries, weighted %				
0	118 (7.3%)	107 (7.1%)	11 (9.3%)	0.18
1–3 times	1427 (82.5%)	1317 (82.1%)	110 (85.9%)	
≥ 4 times	223 (10.3%)	215 (10.9%)	8 (4.8%)	
Oral contraceptive use ≥ 5 years, weighted %	586 (39.0%)	528 (38.3%)	58 (45.4%)	0.11
Lifetime number of male sex partners, weighted %				
≤ 2	480 (26.3%)	454 (26.5%)	26 (24.4%)	0.44
3–4	316 (18.8%)	289 (18.3%)	27 (24.1%)	
5–9	448 (27.1%)	415 (27.5%)	33 (23.1%)	
≥ 10	437 (27.8%)	400 (27.7%)	37 (28.4%)	
Age at first sexual intercourse, weighted %				
≤ 16	769 (45.4%)	714 (45.1%)	55 (48.7%)	0.30
17–18	478 (28.3%)	436 (28.0%)	42 (31.2%)	
≥ 19	441 (26.3%)	415 (26.9%)	26 (20.2%)	
Number of vaginal or anal sex in the past 12 months, weighted % (2005–2006)				
0–11 times	185 (22.1%)	169 (22.3%)	16 (20.7%)	0.001
12–51 times	293 (39.5%)	264 (37.3%)	29 (63.8%)	
52–103 times	185 (22.4%)	179 (23.2%)	6 (12.6%)	
≥ 104 times	139 (16.0%)	136 (17.2%)	3 (2.9%)	
High-risk alcohol intake, weighted %	129 (8.1%)	120 (8.0%)	9 (8.7%)	0.85
Smoking status, weighted %				
Never smoker	1060 (55.3%)	994 (56.3%)	66 (45.7%)	0.02
Ex-smoker	292 (18.1%)	269 (18.1%)	23 (18.3%)	
Current smoker	416 (26.6%)	376 (25.6%)	40 (36.1%)	
Covered by health insurance, weighted %	1342 (80.9%)	1235 (80.3%)	107 (86.1%)	0.06
Number of healthcare utilization in the past year, weighted %				
0–1 time	537 (30.8%)	504 (31.1%)	33 (28.2%)	0.10
2–3 times	438 (27.6%)	414 (28.3%)	24 (21.6%)	
4–9 times	450 (24.2%)	412 (24.1%)	38 (24.4%)	
≥ 10 times	343 (17.4%)	309 (16.5%)	34 (25.9%)	
History of cervical cancer	38 (2.2%)	30 (1.9%)	8 (4.9%)	0.02

Values are presented as numbers (weighted %).

*HPV* human papillomavirus.

P values were calculated using Rao–Scott Chi-square test.

The prevalence of any HPV and high-risk HPV was 42.6% (95% CI 39.6–45.6%) and 26.7% (95% CI 24.0–29.6%), respectively. In the unadjusted analysis, endometriosis diagnosis was not associated with the prevalence of any HPV (PR 0.85, 95% CI 0.60–1.21; Table 2). The fully-adjusted prevalence ratio (aPR) was 0.84 (95% CI 0.61–1.15). In addition, there was no significant association between the diagnosis of endometriosis and high-risk HPV infection in the fully adjusted model (aPR 0.71, 95% CI 0.44–1.14) (Table 3). Furthermore, the associations did not change substantially when we used an indicator variable or multiple imputation to account for missing values (Tables S2 and S3).

**Table 2:** Adjusted prevalence ratios (aPR) of HPV infection by endometriosis.

	Unadjusted	Model 1	Model 2	Model 3
	PR (95% CI)	aPR (95% CI)	aPR (95% CI)	aPR (95% CI)
Endometriosis				
No	Ref	Ref	Ref	Ref
Yes	0.85 (0.60–1.21)	0.87 (0.63–1.20)	0.86 (0.62–1.17)	0.84 (0.61–1.15)
Age categories				
20–34		Ref	Ref	Ref
35–54		0.92 (0.79–1.06)	0.95 (0.81–1.11)	0.94 (0.81–1.08)
Race/ethnicity				
Hispanic		0.97 (0.77–1.22)	1.00 (0.79–1.26)	1.11 (0.89–1.39)
Non-Hispanic White		Ref	Ref	Ref
Non-Hispanic Black		1.30 (1.14–1.48)	1.32 (1.17–1.50)	1.45 (1.28–1.64)
Other races		1.15 (0.85–1.55)	1.13 (0.84–1.52)	1.19 (0.86–1.66)
Ratio of family income to poverty				
< 1.5		Ref	Ref	Ref
1.5 ≤ < 3.0		0.95 (0.81–1.12)	0.92 (0.79–1.07)	0.97 (0.84–1.13)
≥ 3.0		0.77 (0.63–0.94)	0.72 (0.60–0.87)	0.81 (0.68–0.97)
Marital status				
Married/living with partners		Ref	Ref	Ref
Widowed/divorced/separated		1.43 (1.24–1.66)	1.42 (1.22–1.65)	1.38 (1.19–1.60)
Never married		1.41 (1.15–1.72)	1.35 (1.12–1.62)	1.27 (1.06–1.51)
Number of vaginal/cesarean deliveries				
0			Ref	Ref
1–3 times			0.77 (0.60–0.98)	0.77 (0.60–0.99)
≥ 4 times			0.62 (0.43–0.90)	0.61 (0.42–0.89)
Duration of oral contraceptive use				
< 5 years			Ref	Ref
≥ 5 years			1.03 (0.87–1.21)	1.04 (0.88–1.22)
High-risk alcohol intake				
No				Ref
Yes				1.29 (1.04–1.60)
Smoking status				
Never smoker				Ref
Ex-smoker				1.02 (0.82–1.26)
Current smoker				1.37 (1.19–1.58)

*HPV* human papillomavirus.

**Table 3:** Adjusted prevalence ratios (aPR) of high-risk HPV infection by endometriosis.

	Unadjusted	Model 1	Model 2	Model 3
	PR (95% CI)	aPR (95% CI)	aPR (95% CI)	aPR (95% CI)
Endometriosis				
No	Ref	Ref	Ref	Ref
Yes	0.72 (0.45–1.15)	0.75 (0.47–1.20)	0.74 (0.46–1.17)	0.71 (0.44–1.14)
Age categories				
20–34		Ref	Ref	Ref
35–54		0.78 (0.64–0.96)	0.83 (0.67–1.02)	0.82 (0.66–1.00)
Race/ethnicity				
Hispanic		0.89 (0.66–1.20)	0.89 (0.66–1.19)	1.04 (0.79–1.38)
Non-Hispanic White		Ref	Ref	Ref
Non-Hispanic Black		1.17 (0.98–1.41)	1.19 (1.00–1.42)	1.36 (1.15–1.61)
Other races		1.01 (0.65–1.58)	0.98 (0.62–1.53)	1.07 (0.66–1.73)
Ratio of family income to poverty				
< 1.5		Ref	Ref	Ref
1.5 ≤ < 3.0		0.96 (0.78–1.18)	0.93 (0.77–1.13)	1.02 (0.85–1.22)
≥ 3.0		0.81 (0.63–1.04)	0.78 (0.62–0.99)	0.93 (0.74–1.18)
Marital status				
Married/living with partners		Ref	Ref	Ref
Widowed/divorced/separated		1.41 (1.14–1.76)	1.41 (1.14–1.75)	1.35 (1.08–1.68)
Never married		1.63 (1.28–2.08)	1.56 (1.24–1.95)	1.43 (1.15–1.78)
Number of vaginal/cesarean deliveries				
0			Ref	Ref
1–3 times			0.85 (0.62–1.18)	0.85 (0.61–1.19)
≥ 4 times			0.59 (0.36–0.99)	0.58 (0.34–0.99)
Duration of oral contraceptive use				
< 5 years			Ref	Ref
≥ 5 years			0.84 (0.67–1.04)	0.85 (0.68–1.05)
High-risk alcohol intake				
No				Ref
Yes				1.36 (0.94–1.95)
Smoking status				
Never smoker				Ref
Ex-smoker				0.97 (0.71–1.32)
Current smoker				1.62 (1.34–1.96)

*HPV* human papillomavirus.

Overall, there were 38 participants with a history of cervical cancer (Table 1). Women with endometriosis were more likely to have a history of cervical cancer (PR 2.55, 95% CI 1.18–5.50); however, the result was not significant after adjustment (aPR 2.31, 95% CI 0.95–5.60) (Table S4).

**Figure 1:**
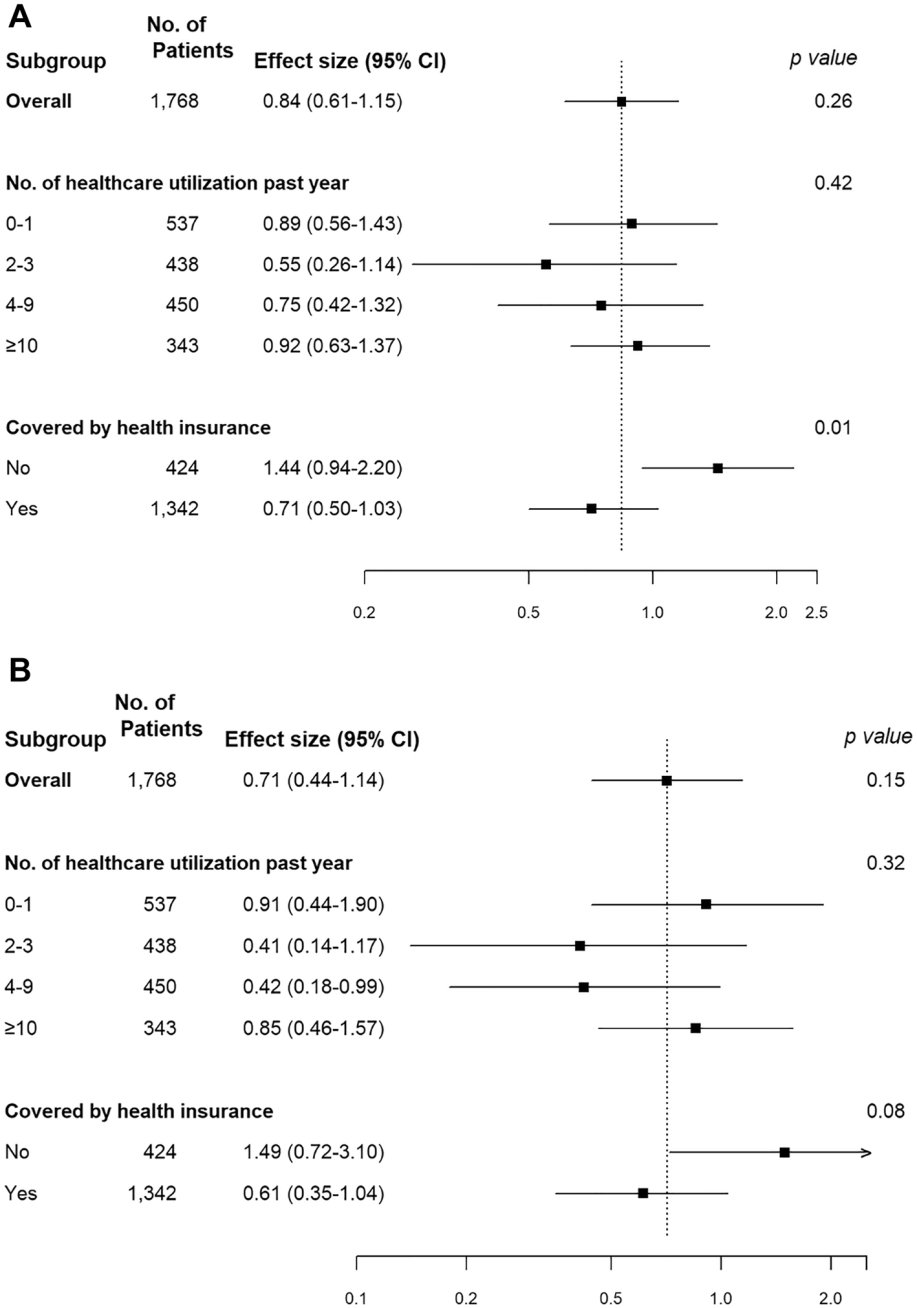
Subgroup analysis of the association between endometriosis and human papillomavirus (HPV) infection by healthcare utilization or health insurance coverage. (**A**) Any HPV infection. (**B**) High-risk HPV infection.

In subgroup analyses by the categories of the number of healthcare utilization in the past year, the association between endometriosis diagnosis and the prevalence of any HPV or high-risk HPV infection was similar across (P for interaction = 0.42 and 0.32, respectively). On the other hand, in a subgroup analysis by health insurance coverage, women with endometriosis had a lower prevalence of HPV infection compared to those without endometriosis only among participants with health insurance (aPR 0.71, 95% CI 0.50–1.03; P for interaction = 0.01; Fig. 1). The findings were similar for high-risk HPV infection, but the interaction was marginally significant (P = 0.08).

## Discussion

In this nationally representative study of HPV vaccine-naïve participants, we found that endometriosis diagnosis was not associated with the prevalence of any HPV or high-risk HPV in women of reproductive age. However, women with endometriosis had a lower prevalence of HPV among those with health insurance. To our knowledge, this is the first study that investigated the association between endometriosis and HPV infection in a nationwide survey.

As yet, a definite biological mechanism between endometriosis and HPV infection has not been proven. HPV is a sexually transmitted disease and women with endometriosis may have a low prevalence of HPV because of decreased sexual activities due to dyspareunia and pelvic pain^8,12,15^. On the other hand, almost all types of immune cells demonstrated abnormalities in endometriosis^18,25^, including the reduced function of natural killer cells^17^, and decreased proportion of peripheral regulatory T cells^21^. In addition, other immunologic factors, such as increased inflammatory activity in peritoneal fluid and reduced immune surveillance, were found in endometriosis^16^. Therefore, it may be hypothesized that abnormal immune function may promote not only endometriotic cell maintenance and proliferation but also HPV infection, although there is no robust evidence. However, our study found no clear association between endometriosis diagnosis and HPV infection after accounting for multiple sociodemographic factors. The prevalence of high-risk HPV was also similar regardless of endometriosis diagnosis. In another study, the prevalence of any type of HPV infection was similar between surgically confirmed endometriosis patients and healthy controls^24^. In other studies, however, high-risk HPV was more prevalent in women with endometriosis compared to those without endometriosis^22,23^. The inconsistent results may be due to small sample sizes, different study populations, and HPV detection methods. Furthermore, they did not perform any adjustment for potential confounders affecting endometriosis diagnosis and HPV infection.

In this study, we were also able to evaluate sociodemographic and behavioral factors associated with endometriosis and HPV infection. For instance, women with endometriosis were more likely to be covered by health insurance (86.1% vs. 80.3%, P = 0.06) and had more healthcare utilization than women without endometriosis (Table 1). When stratified, endometriosis diagnosis was associated with a lower prevalence of HPV infection in women with health insurance but with a higher prevalence in those without health insurance (Fig. 1). Better access to healthcare may involve education on safer sex practices, and thus lower HPV infection^26^. In contrast, the association between endometriosis diagnosis and HPV infection did not differ by the number of healthcare utilizations. However, healthcare utilization was not distinguished by specialty in the NHANES.

As chronic pelvic pain and dyspareunia accompanied by endometriosis reduce sexual activity^8,12^, we evaluated the association between endometriosis and HPV infection stratified by the frequency of sexual activity (“in the past 12 months, about how many times have you had vaginal or anal sex?”) included in the 2005–2006 NHANES cycle. Women with endometriosis were less likely to be involved in frequent sexual activity (52–103 times or ≥ 104 times in the past 12 months) (Table S5). However, the association between endometriosis and HPV infection did not differ by the frequency of sexual activity (P for interaction > 0.05) (Supplementary Fig. 1). In addition, adjusting for the frequency of vaginal or anal sex in the past year did not change the association between endometriosis diagnosis and the prevalence of any HPV or high-risk HPV substantially (Tables S6 and S7).

Although women with endometriosis did not demonstrate a higher prevalence of HPV compared to those without endometriosis, we found that the prevalence of cervical cancer was 2.31 times higher in women with endometriosis than in women without endometriosis. There are several reasons that may explain the difference in the associations of endometriosis with HPV infection and cervical cancer. First, HPV infection status was assessed as part of the survey whereas a history of cervical cancer was based on a physician’s diagnosis. Women with endometriosis are likely to be under more frequent surveillance for cervical cancer than women without endometriosis, which would increase the detection of cervical cancer (surveillance bias). In this case, women with endometriosis may have been diagnosed with cervical cancer at an earlier stage because they have more frequent visits to healthcare providers. However, the information about the cervical cancer stage at initial diagnosis or treatment modality was not available in the NHANES and could not be compared. Similarly, the increased hazard ratio of cervical cancer was found in women with endometriosis compared to the general population in a Scottish nationwide cohort^27^, but early diagnosis of cervical cancer in women with endometriosis has never been reported in the previous cohort studies^10–12,27^. Second, HPV infection may be self-limited and measurement on a single instance may not reflect persistent infection which leads to the development of cervical cancer.

Our study has several limitations. First, the diagnosis of endometriosis is commonly delayed by 4–10 years from the onset of symptoms^28^, and HPV infection does not cause any noticeable symptoms or signs. Subsequently, most people who have either endometriosis or HPV infection will not know whether they have these diseases. Therefore, misclassification of both diseases may be common. Moreover, the presence of endometriosis was exclusively based on a self-report of physician diagnosis and is subject to misclassification. False statements of endometriosis diagnosis were reported to occur frequently^29^. However, the prevalence of endometriosis in the present study (8.9%) was comparable to that of the previous report^1^. Furthermore, a few studies reported the validity of self-reported endometriosis and it was relatively accurate (ranging from 72 to 95%)^30,31^. Second, the NHANES is a cross-sectional study; therefore, the null association between endometriosis and HPV infection in our study should be interpreted with caution. However, a prospective study might not provide a better understanding than a cross-sectional study due to the misclassification stated above. Third, we used data from 2003 to 2006, which may not represent the general population after the introduction and approval of HPV vaccines in the US in 2006. However, HPV vaccination would affect the prevalence of HPV infection and would make the association between endometriosis and HPV infection less interpretable. Because women with endometriosis are more likely to access health care services, they are more likely to receive HPV vaccination and the association between endometriosis and HPV infection may be biased. Finally, there were participants with missing information on potential confounders. However, the results did not change substantially when multiple imputation was applied (Tables S2 and S3).

Despite the limitations, our study population included 1768 women in whom HPV infection was tested with a credible method. The characteristics of the representative survey make the study findings generalizable to the general US women of reproductive age. In addition, we were able to account for several important potential confounders, such as the ratio of family income to poverty, and consider sociodemographic factors, such as health insurance coverage and healthcare utilization, which had not been evaluated previously.

In summary, there was no association between endometriosis and HPV infection in this nationally representative study of HPV vaccine-naïve women of reproductive age. The association was not different by the type of HPV or after adjustment for potential confounders. However, access to healthcare may modify the association between endometriosis and HPV infection. Further investigation in large-scale prospective studies is needed to confirm these findings.

## Materials and methods

### Study population

**Figure 2:**
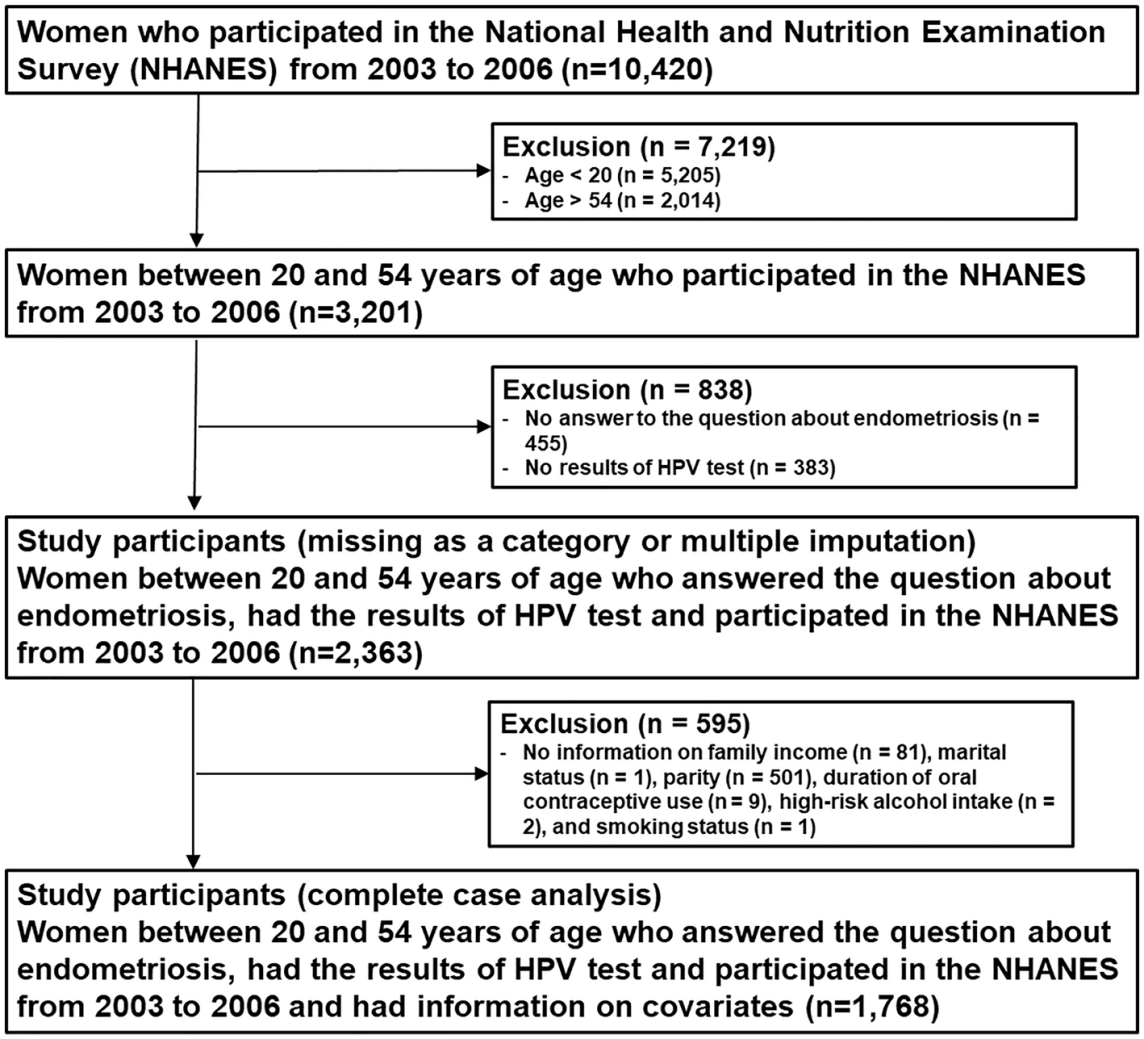
Flow chart of study participants.

The NHANES is a multistage, complex survey of a representative sample of the non-institutionalized US population, which includes patient interviews, physical examinations, and laboratory tests. From 1999 to 2006, female participants between 20 and 54 years of age were asked about a diagnosis of endometriosis. Since 2003, a vaginal swab for genital HPV has been performed in females of 18–59 years old. Of the 10,420 female participants in the NHANES from 2003 to 2006, there were 3201 participants between 20 and 54 years of age (Fig. 2). We excluded participants (n = 838) who had missing information on a diagnosis of endometriosis (n = 455) or on genital HPV (n = 383). For the main analysis, a total of 1738 participants were included after further excluding 595 participants who did not have information on family income (n = 81), marital status (n = 1), parity (n = 501), duration of oral contraceptive use (n = 9), high-risk alcohol intake (n = 2), or smoking status (n = 1).

### Ethics approval and informed consent

The National Center for Health Statistics (NCHS) Research Ethics Review Board approved the survey protocol and data collection methods of the NHANES (Protocol#98-12, and Protocol#2005-06). All study participants provided informed consent before participation. The institutional review board of the Seoul National University Hospital waived an ethical approval due to the retrospective nature of the study using de-identified and publicly available data.

### Diagnosis of endometriosis

Diagnosis of endometriosis was based on a question included as part of the reproductive health questionnaire administered to 20–54 year old females from 1999 to 2006: “Has a doctor or other health professional ever told you that you had endometriosis?”. Those who answered as having endometriosis were further asked about the age at first diagnosis.

### Genital HPV test

A cervicovaginal sample was self-collected in 18–59 years old females in the mobile examination center (MEC), and specimens were mailed within one week to the Centers for Disease Control and Prevention (CDC) laboratory for processing^32,33^. A total of 37 HPV DNA genotypes were determined using the Research Use Only Linear Array HPV Genotyping Test (Roche Molecular Diagnostics, Indianapolis, IN). Samples were considered positive for HPV if any of the 37 HPV types were identified, including high-risk (16, 18, 26, 31, 33, 35,39, 45, 51, 52, 53, 56, 58, 59, 66, 68, 73, and 82) and low-risk (6, 11, 40, 42, 54, 55, 61, 62, 64, 67, 69, 70, 71, 72, 81, 82, 83, 84, 89, and IS39) types^34^. If samples tested negative for both HPV and β-globin (negative control for sample amplification), they were considered inadequate and were excluded from the analysis^35^.

Participants who were positive for HPV, were further categorized as high-risk HPV (+) if they were positive for any of the high-risk HPV types. Those who tested positive for only low-risk HPV types were classified as high-risk HPV (−).

### Other covariates

Demographic variables, including age, race/ethnicity, ratio of family income to poverty, and marital status, were collected through household interviews. Race/ethnicity was categorized as Hispanic, Non-Hispanic White, Non-Hispanic Black, and other races. A ratio of family income to poverty was categorized as < 150%, 150 to less than 300%, and ≥ 300%.

Reproductive history and sexual history were asked at the MEC by a trained interviewer^35^. Parity (a total number of vaginal or Cesarean deliveries) was based on self-report and was categorized as 0, 1–3, and ≥ 4. The use and duration of oral contraceptives were also self-reported. Duration of oral contraceptive use was further categorized as < 5 years and ≥ 5 years. The number of vaginal or anal sex in the past 12 months (never, once, 2–11 times, 12–51 times, 52–103 times, 104–364 times, and 365 times or more in the past 12 months) was available only in the NHANES 2005–2006 cycle. It was further converted to the average frequency of vaginal or anal sex per year: 0–11 times per year (approximately less than once a month), 12–51 times per year (once a month to less than once a week), 52–103 times per year (1–2 times a week), and ≥ 104 times per year (2 or more times a week). A history of cervical cancer was based on a self-report of a physician’s diagnosis.

High-risk alcohol intake was defined as four or more drinks every day. Whether a participant had smoked at least 100 cigarettes in their entire life was asked and smoking status was further categorized as never smoker, ex-smoker, and current smoker. Information on health insurance coverage and the number of healthcare visits in the prior 12 months was also collected.

### Statistical analysis

In all analyses, appropriate variance estimation and sampling weights were used to account for the complex sample design of the NHANES and oversampling, nonresponse, and post-stratification^36^.

Participant characteristics are summarized by the number of participants (weighted proportion) and the mean (standard error) for categorical and continuous variables, respectively. They were compared by the presence of endometriosis using Rao–Scott Chi-square tests or univariable linear regression, as appropriate.

The primary outcome was the prevalence of any HPV infection. The secondary outcome was the prevalence of high-risk HPV infection. We estimated the prevalence ratios (PRs) and corresponding 95% confidence intervals (CI) of HPV infection in participants with and without endometriosis using multivariable Poisson regression models. We used progressive degrees of adjustment. Model 1 was adjusted for age, race/ethnicity, the ratio of family income to poverty, and marital status; Model 2 was further adjusted for parity and duration of oral contraceptive use; Model 3 was further adjusted for high-risk alcohol intake and smoking status. The covariates selected for adjustment were considered to be related to both endometriosis and HPV infection in previous literature^37–41^. We additionally performed subgroup analysis by the number of healthcare utilizations in the past year and by health insurance coverage.

For main analysis, complete-case analyses were performed. To account for missing data, we performed two additional analyses including 2363 participants (Fig. 2). First, we performed the analysis by classifying missing value as a separate category. Second, missing data were handled using ten datasets generated by multiple imputation by chained equations. The results using 10 individual data sets were combined and the pooled estimates were estimated using the methods by Little and Rubin^42^.

We used Stata 17.0 (StataCorp LLC, College Station, TX) for all statistical analyses. All P values < 0.05 were considered significant.

## Supplementary Information

Supplementary Information.

## Data Availability

The de-identified data is available from NHANES website (https://wwwn.cdc.gov/nchs/nhanes/default.aspx).
