# Supplementary material for: Association of endometriosis with genital human papillomavirus infection in US women: a national population-based study
Source: Sci Rep. 2023 May 17;13:8020. doi: 10.1038/s41598-023-35153-0 (PMC10192320; doi:10.1038/s41598-023-35153-0)
Supplement: Supplementary file 1 — Supplementary Information. [file 41598_2023_35153_MOESM1_ESM.pdf]

Supplementary Table 1. Baseline characteristics of study participants (including participants with missing on any covariate)

Variables	All	Women without Endometriosis	Women with Endometriosis	P value
No. of participants	2,363	2,194	169	
No. of participants, weighted	59,890,321	54,521,954	5,368,367	
HPV (+)	1,110 (43.7%)	1,034 (44.1%)	76 (40.0%)	0.49
High risk HPV (+)	714 (28.0%)	673 (28.5%)	41 (22.7%)	0.46
Age categories, weighted %				
20-34	1,117 (38.9%)	1,080 (41.0%)	37 (17.9%)	<0.001
35-54	1,246 (61.1%)	1,114 (59.0%)	132 (82.1%)	
Race/Ethnicity, weighted %				
Hispanic	585 (12.5%)	570 (13.3%)	15 (4.4%)	<0.001
Non-Hispanic White	1,117 (69.2%)	1,007 (67.9%)	110 (82.7%)	
Non-Hispanic Black	556 (12.9%)	516 (13.1%)	40 (10.3%)	
Other races	105 (5.4%)	101 (5.7%)	4 (2.6%)	
Education, weighted %				
Less than high school	509 (13.6%)	489 (14.0%)	20 (9.6%)	0.19
High school graduate	522 (22.7%)	484 (22.1%)	38 (28.6%)	
Some college or AA degree	810 (37.0%)	746 (37.2%)	64 (35.8%)	
College graduate or above	521 (26.6%)	474 (26.7%)	47 (26.0%)	

Missing	1 (0.05%)	1 (0.05%)	0	
Ratio of family income to poverty, weighted %				
< 1.5	782 (23.3%)	742 (23.5%)	40 (20.9%)	0.16
1.5 ≤ < 3.0	548 (23.3%)	518 (23.8%)	30 (18.2%)	
≥ 3.0	952 (50.3%)	856 (49.5%)	96 (58.4%)	
Missing	81 (3.1%)	78 (3.2%)	3 (2.5%)	
Marital status, weighted %				
Married/ living with partners	1,482 (65.0%)	1,380 (65.2%)	102 (63.3%)	0.03
Widowed/ divorced/ separated	363 (15.7%)	323 (15.0%)	40 (23.1%)	
Never married	517 (19.2%)	490 (19.7%)	27 (13.6%)	
Missing	1 (0.1%)	1 (0.1%)	0	
Number of vaginal/ Cesarean deliveries, weighted %				
0	120 (5.4%)	109 (5.2%)	11 (7.2%)	0.14
1-3 times	1,479 (62.6%)	1,366 (61.9%)	113 (70.1%)	
≥ 4 times	239 (7.9%)	231 (8.3%)	8 (3.8%)	
Missing	525 (24.1%)	488 (24.6%)	37 (18.9%)	
Duration of oral contraceptive use, weighted %				
< 5 years	1,591 (61.9%)	1,502 (62.9%)	89 (51.4%)	0.01
≥ 5 years	761 (37.8%)	683 (36.9%)	78 (46.8%)	

Missing	11 (0.4%)	9 (0.2%)	2 (1.9%)	
Lifetime number of male sex partners, weighted %				
≤ 2	648 (25.9%)	614 (26.1%)	34 (23.5%)	0.69
3-4	413 (17.2%)	380 (17.0%)	33 (19.4%)	
5-9	584 (25.4%)	538 (25.6%)	46 (23.2%)	
≥ 10	564 (25.8%)	518 (25.7%)	46 (27.6%)	
Missing	154 (5.7%)	144 (5.7%)	10 (6.3%)	
Age at first sexual intercourse (years), weighted %				
≤ 16	955 (40.6%)	885 (40.4%)	70 (43.3%)	0.21
17-18	626 (27.0%)	572 (26.7%)	54 (29.8%)	
≥ 19	641 (27.2%)	605 (27.8%)	36 (21.5%)	
Missing	141 (5.1%)	132 (5.1%)	9 (5.5%)	
Number of vaginal or anal sex in the past 12 months, weighted % (2005-2006)				
0-11 times	244 (18.9%)	221 (18.9%)	23 (19.2%)	<0.001
12-51 times	379 (32.4%)	341 (31.4%)	38 (42.3%)	
52-103 times	242 (19.0%)	233 (20.0%)	9 (9.2%)	
≥ 104 times	181 (13.3%)	176 (14.3%)	5 (3.2%)	
Missing	227 (16.4%)	206 (15.4%)	21 (26.0%)	
High-risk alcohol intake, weighted %				

No	2,198 (92.4%)	2,041 (92.5%)	157 (91.5%)	0.76
Yes	163 (7.5%)	151 (7.4%)	12 (8.5%)	
Missing	2 (0.1%)	2 (0.1%)	0	
Smoking status, weighted %				
Never smoker	1,445 (57.6%)	1,355 (58.5%)	90 (48.1%)	0.02
Ex-smoker	379 (16.8%)	349 (16.8%)	30 (17.5%)	
Current smoker	538 (25.6%)	489 (24.7%)	49 (34.4%)	
Missing	1 (0.03%)	1 (0.03%)	0	
Covered by health insurance, weighted %				
No	559 (19.0%)	534 (19.6%)	25 (13.4%)	0.06
Yes	1,797 (80.7%)	1,654 (80.2%)	143 (85.4%)	
Missing	7 (0.3%)	6 (0.2%)	1 (1.1%)	
Number of healthcare utilization in the past year, weighted %				
0-1 time	723 (31.0%)	683 (31.5%)	40 (25.4%)	0.04
2-3 times	621 (29.6%)	583 (30.1%)	38 (25.1%)	
4-9 times	590 (23.5%)	540 (23.3%)	50 (25.7%)	
≥ 10 times	428 (15.8%)	387 (15.1%)	41 (23.8%)	
Missing	1 (0.05%)	1 (0.05%)	0	
History of cervical cancer				

No	2,323 (98.2%)	2,163 (98.5%)	160 (95.5%)	0.01
Yes	40 (1.8%)	31 (1.5%)	9 (4.5%)	

Values are presented as numbers (weighted %).

HPV, human papillomavirus

P values were calculated using Rao-Scott chi-square test

Supplementary Table 2. Adjusted prevalence ratios (aPR) of human papillomavirus (HPV) infection by endometriosis

	Complete case analysis (n=1,768)	Missing as a category (n=2,363)	Multiple imputation (n=2,363)
	aPR (95% CI)	aPR (95% CI)	aPR (95% CI)
Endometriosis			
No	Ref	Ref	Ref
Yes	0.84 (0.61-1.15)	0.90 (0.69-1.19)	0.89 (0.68-1.17)
Age categories			
20-34	Ref	Ref	Ref
35-54	0.94 (0.81-1.08)	0.88 (0.77-1.00)	0.88 (0.77-1.01)
Race/Ethnicity			
Hispanic	1.11 (0.89-1.39)	1.11 (0.95-1.31)	1.11 (0.95-1.30)
Non-Hispanic White	Ref	Ref	Ref
Non-Hispanic Black	1.45 (1.28-1.64)	1.42 (1.29-1.57)	1.40 (1.28-1.53)
Other races	1.19 (0.86-1.66)	0.98 (0.72-1.34)	0.98 (0.71-1.33)
Ratio of family income to poverty			
< 1.5	Ref	Ref	Ref
1.5 ≤ < 3.0	0.97 (0.84-1.13)	0.98 (0.85-1.13)	0.96 (0.84-1.11)
≥ 3.0	0.81 (0.68-0.97)	0.84 (0.73-0.96)	0.82 (0.71-0.94)
Missing		1.00 (0.77-1.30)	
Marital status			
Married/ living with partners	Ref	Ref	Ref
Widowed/ divorced/ separated	1.38 (1.19-1.60)	1.39 (1.19-1.63)	1.39 (1.19-1.62)

Never married	1.27 (1.06-1.51)	1.21 (1.05-1.39)	1.22 (1.07-1.40)
Missing		3.13 (2.65-3.69)	
Number of vaginal/ Cesarean deliveries			
0	Ref	Ref	Ref
1-3 times	0.77 (0.60-0.99)	0.79 (0.61-1.02)	0.76 (0.60-0.97)
≥ 4 times	0.61 (0.42-0.89)	0.65 (0.44-0.96)	0.60 (0.43-0.84)
Missing		0.86 (0.66-1.11)	
Duration of oral contraceptive use			
< 5 years	Ref	Ref	Ref
≥ 5 years	1.04 (0.88-1.22)	1.11 (0.96-1.28)	1.10 (0.96-1.26)
Missing		0.67 (0.34-1.32)	
High-risk alcohol intake			
No	Ref	Ref	Ref
Yes	1.29 (1.04-1.60)	1.20 (0.99-1.45)	1.20 (0.99-1.45)
Missing		1.69 (1.19-2.40)	
Smoking status			
Never smoker	Ref	Ref	Ref
Ex-smoker	1.02 (0.82-1.26)	1.09 (0.91-1.30)	1.09 (0.90-1.30)
Current smoker	1.37 (1.19-1.58)	1.41 (1.24-1.59)	1.40 (1.23-1.59)
Missing		2.47 (2.13-2.87)	

All models were adjusted for age, race/ethnicity, the ratio of family income to poverty, marital status, parity, duration of oral contraceptive use, high-risk alcohol intake, and smoking status.

Supplementary Table 3. Adjusted prevalence ratios (aPR) of high-risk human papillomavirus (HPV) infection by endometriosis

	Complete case analysis (n=1,768)	Missing as a category (n=2,363)	Multiple imputation (n=2,363)
	aPR (95% CI)	aPR (95% CI)	aPR (95% CI)
Endometriosis			
No	Ref	Ref	Ref
Yes	0.71 (0.44-1.14)	0.81 (0.53-1.23)	0.79 (0.52-1.21)
Age categories			
20-34	Ref	Ref	Ref
35-54	0.82 (0.66-1.00)	0.81 (0.67-0.98)	0.82 (0.67-0.99)
Race/Ethnicity			
Hispanic	1.04 (0.79-1.38)	0.97 (0.79-1.21)	0.98 (0.78-1.23)
Non-Hispanic White	Ref	Ref	Ref
Non-Hispanic Black	1.36 (1.15-1.61)	1.33 (1.14-1.55)	1.31 (1.11-1.56)
Other races	1.07 (0.66-1.73)	0.96 (0.64-1.46)	0.96 (0.63-1.46)
Ratio of family income to poverty			
< 1.5	Ref	Ref	Ref
1.5 ≤ < 3.0	1.02 (0.85-1.22)	1.03 (0.85-1.25)	1.01 (0.83-1.23)
≥ 3.0	0.93 (0.74-1.18)	0.91 (0.77-1.08)	0.89 (0.75-1.06)
Missing		1.21 (0.90-1.64)	
Marital status			
Married/ living with partners	Ref	Ref	Ref
Widowed/ divorced/ separated	1.35 (1.08-1.68)	1.33 (1.06-1.67)	1.33 (1.05-1.67)

Never married	1.43 (1.15-1.78)	1.35 (1.15-1.58)	1.36 (0.35-0.92)
Missing		5.02 (4.15-6.08)	
Number of vaginal/ Cesarean deliveries			
0	Ref	Ref	Ref
1-3 times	0.85 (0.61-1.19)	0.87 (0.63-1.21)	0.87 (0.63-1.19)
≥ 4 times	0.58 (0.34-0.99)	0.60 (0.35-1.03)	0.56 (0.35-0.92)
Missing		0.90 (0.67-1.22)	
Duration of oral contraceptive use			
< 5 years	Ref	Ref	Ref
≥ 5 years	0.85 (0.68-1.05)	0.92 (0.75-1.12)	0.91 (0.74-1.11)
Missing		0.24 (0.04-1.54)	
High-risk alcohol intake			
No	Ref	Ref	Ref
Yes	1.36 (0.94-1.95)	1.26 (0.90-1.75)	1.25 (0.90-1.75)
Missing		N/A	
Smoking status			
Never smoker	Ref	Ref	Ref
Ex-smoker	0.97 (0.71-1.32)	1.12 (0.89-1.42)	1.12 (0.88-1.43)
Current smoker	1.62 (1.34-1.96)	1.60 (1.34-1.90)	1.59 (1.33-1.90)
Missing		4.18 (3.41-5.12)	

All models were adjusted for age, race/ethnicity, the ratio of family income to poverty, marital status, parity, duration of oral contraceptive use, high-risk alcohol intake, and smoking status.

Supplementary Table 4. Adjusted prevalence ratios (aPR) of cervical cancer by endometriosis

	Unadjusted	Model 1	Model 2	Model 3
	PR (95% CI)	aPR (95% CI)	aPR (95% CI)	aPR (95% CI)
Endometriosis				
No	Ref	Ref	Ref	Ref
Yes	2.55 (1.18-5.50)	2.10 (0.99-4.48)	2.42 (1.05-5.57)	2.31 (0.95-5.60)
Age categories				
20-34		Ref	Ref	Ref
35-54		0.85 (0.41-1.73)	0.69 (0.33-1.44)	0.70 (0.34-1.41)
Race/Ethnicity				
Hispanic		0.27 (0.09-0.88)	0.26 (0.08-0.82)	0.45 (0.14-1.51)
Non-Hispanic White		Ref	Ref	Ref
Non-Hispanic Black		0.36 (0.11-1.13)	0.32 (0.10-1.01)	0.47 (0.13-1.70)
Other races		0.93 (0.21-4.18)	1.02 (0.22-4.71)	1.21 (0.25-5.81)
Ratio of family income to poverty				
< 1.5		Ref	Ref	Ref
1.5 ≤ < 3.0		0.26 (0.08-0.82)	0.29 (0.09-0.92)	0.31 (0.09-1.11)
≥ 3.0		0.30 (0.14-0.66)	0.34 (0.14-0.81)	0.43 (0.18-1.02)
Marital status				
Married/ living with partners		Ref	Ref	Ref
Widowed/ divorced/ separated		1.53 (0.72-3.24)	1.60 (0.78-3.26)	1.45 (0.74-2.84)
Never married		0.55 (0.12-2.66)	0.59 (0.12-2.86)	0.46 (0.10-2.15)

Number of vaginal/ Cesarean deliveries		
0	Ref	Ref
1-3 times	1.22 (0.15-10.22)	1.38 (0.17-11.35)
≥ 4 times	3.65 (0.31-43.12)	3.96 (0.31-51.05)
Duration of oral contraceptive use		
< 5 years	Ref	Ref
≥ 5 years	1.42 (0.67-3.00)	1.37 (0.64-2.95)
High-risk alcohol intake		
No		Ref
Yes		0.76 (0.25-2.36)
Smoking status		
Never smoker		Ref
Ex-smoker		5.07 (1.58-16.25)
Current smoker		5.44 (1.79-16.58)

Supplementary Table 5. Multivariable ordinal logistic regression model of the relationship between endometriosis and the number of vaginal or anal sex in the past 12 months (2005-2006)

	≤ 11	12-51	52-103	≥104
Endometriosis				
No	Ref	Ref	Ref	Ref
Yes	Ref	1.29 (0.67-2.50)	0.33 (0.13-0.84)	0.19 (0.05-0.69)
Age categories				
20-34	Ref	Ref	Ref	Ref
35-54	Ref	0.33 (0.22-0.49)	0.44 (0.25-0.78)	0.36 (0.23-0.57)
Race/Ethnicity				
Hispanic	Ref	0.59 (0.31-1.14)	1.19 (0.76-1.85)	1.22 (0.53-2.80)
Non-Hispanic White	Ref	Ref	Ref	Ref
Non-Hispanic Black	Ref	0.61 (0.34-1.07)	0.79 (0.50-1.24)	0.81 (0.36-1.80)
Other races	Ref	1.27 (0.47-3.40)	1.01 (0.56-1.82)	1.05 (0.46-2.40)
Ratio of family income to poverty				
< 1.5	Ref	Ref	Ref	Ref
1.5 ≤ < 3.0	Ref	1.14 (0.62-2.09)	1.22 (0.78-1.90)	1.93 (1.35-2.76)
≥ 3.0	Ref	0.94 (0.53-1.66)	0.71 (0.41-1.25)	1.21 (0.51-2.87)
Marital status				
Married/ living with partners	Ref	Ref	Ref	Ref
Widowed/ divorced/ separated	Ref	0.57 (0.28-1.15)	0.69 (0.31-1.53)	0.78 (0.39-1.56)
Never married	Ref	0.28 (0.16-0.48)	0.31 (0.14-0.67)	0.50 (0.22-1.14)
Number of vaginal/ Cesarean deliveries				
0	Ref	Ref	Ref	Ref

1-3 times	Ref	0.81 (0.34-1.91)	1.02 (0.33-3.15)	4.34 (1.08-17.44)
≥ 4 times	Ref	1.27 (0.40-4.05)	1.64 (0.49-5.54)	3.83 (0.62-23.83)
Duration of oral contraceptive use				
< 5 years	Ref	Ref	Ref	Ref
≥ 5 years	Ref	1.13 (0.67-1.91)	1.26 (0.73-2.16)	1.26 (0.85-1.87)
High-risk alcohol intake				
No	Ref	Ref	Ref	Ref
Yes	Ref	1.27 (0.56-2.90)	0.88 (0.40-1.94)	1.61 (0.72-3.59)
Smoking status				
Never smoker	Ref	Ref	Ref	Ref
Ex-smoker	Ref	0.90 (0.63-1.28)	1.25 (0.74-2.12)	1.00 (0.46-2.14)
Current smoker	Ref	1.15 (0.69-1.90)	1.39 (0.87-2.22)	0.91 (0.45-1.84)

Supplementary Table 6. Adjusted prevalence ratios (aPR) of human papillomavirus (HPV) infection by endometriosis (2005-2006)

	Model 3	Model 3 and further adjusted for the frequency of sexual activity
	aPR (95% CI)	aPR (95% CI)
Endometriosis		
No	Ref	Ref
Yes	0.65 (0.37-1.14)	0.76 (0.46-1.27)
Age categories		
20-34	Ref	Ref
35-54	0.96 (0.75-1.23)	0.97 (0.77-1.24)
Race/Ethnicity		
Hispanic	0.97 (0.66-1.45)	1.00 (0.65-1.55)
Non-Hispanic White	Ref	Ref
Non-Hispanic Black	1.56 (1.32-1.84)	1.52 (1.29-1.79)
Other races	1.04 (0.55-1.97)	0.83 (0.42-1.66)
Ratio of family income to poverty		
< 1.5	Ref	Ref
1.5 ≤ < 3.0	1.06 (0.86-1.31)	1.09 (0.91-1.30)
≥ 3.0	0.92 (0.68-1.23)	1.00 (0.74-1.36)
Marital status		
Married/ living with partners	Ref	Ref
Widowed/ divorced/ separated	1.46 (1.13-1.89)	1.54 (1.12-2.12)
Never married	1.28 (1.02-1.62)	1.54 (1.19-2.01)

Number of vaginal/ Cesarean deliveries		
0	Ref	Ref
1-3 times	0.72 (0.46-1.11)	0.78 (0.46-1.32)
≥ 4 times	0.54 (0.28-1.05)	0.56 (0.25-1.28)
Duration of oral contraceptive use		
< 5 years	Ref	Ref
≥ 5 years	1.07 (0.83-1.38)	0.97 (0.72-1.30)
High-risk alcohol intake		
No	Ref	Ref
Yes	1.30 (1.01-1.66)	1.41 (1.10-1.81)
Smoking status		
Never smoker	Ref	Ref
Ex-smoker	1.08 (0.80-1.46)	1.15 (0.81-1.63)
Current smoker	1.61 (1.32-1.95)	1.62 (1.23-2.14)
Number of sexual intercourses in the past year		
≤ 11 times		Ref
12-51 times		0.95 (0.71-1.26)
52-103 times		1.01 (0.70-1.45)
≥ 104 times		0.99 (0.80-1.23)

Supplementary Table 7. Adjusted prevalence ratios (aPR) of high-risk human papillomavirus (HPV) infection by endometriosis (2005-2006)

	Model 3	Model 3 and further adjusted for the frequency of sexual activity
	aPR (95% CI)	aPR (95% CI)
Endometriosis		
No	Ref	Ref
Yes	0.66 (0.31-1.41)	0.82 (0.42-1.62)
Age categories		
20-34	Ref	Ref
35-54	0.96 (0.70-1.30)	1.00 (0.73-1.38)
Race/Ethnicity		
Hispanic	0.88 (0.54-1.44)	0.91 (0.55-1.51)
Non-Hispanic White	Ref	Ref
Non-Hispanic Black	1.52 (1.28-1.81)	1.50 (1.23-1.82)
Other races	0.93 (0.43-2.02)	0.89 (0.45-1.77)
Ratio of family income to poverty		
< 1.5	Ref	Ref
1.5 ≤ < 3.0	0.93 (0.74-1.17)	0.98 (0.77-1.24)
≥ 3.0	0.93 (0.66-1.30)	0.93 (0.68-1.29)
Marital status		
Married/ living with partners	Ref	Ref
Widowed/ divorced/ separated	1.32 (0.95-1.84)	1.52 (1.05-2.20)
Never married	1.37 (1.05-1.79)	1.72 (1.26-2.35)

Number of vaginal/ Cesarean deliveries		
0	Ref	Ref
1-3 times	0.82 (0.47-1.43)	0.86 (0.49-1.53)
≥ 4 times	0.53 (0.23-1.22)	0.55 (0.22-1.34)
Duration of oral contraceptive use		
< 5 years	Ref	Ref
≥ 5 years	0.86 (0.59-1.26)	0.86 (0.57-1.29)
High-risk alcohol intake		
No	Ref	Ref
Yes	1.56 (1.04-2.34)	1.70 (1.10-2.61)
Smoking status		
Never smoker	Ref	Ref
Ex-smoker	0.87 (0.65-1.17)	0.97 (0.75-1.25)
Current smoker	1.67 (1.28-2.18)	1.49 (1.12-2.00)
Number of sexual intercourses in the past year		
≤ 11 times		Ref
12-51 times		0.93 (0.63-1.36)
52-103 times		1.03 (0.70-1.50)
≥ 104 times		0.87 (0.55-1.38)

Supplementary Figure 1. Subgroup analysis in the association between endometriosis and human papillomavirus (HPV) infection by the frequency of sexual activity in the past 12 months (2005-2006)

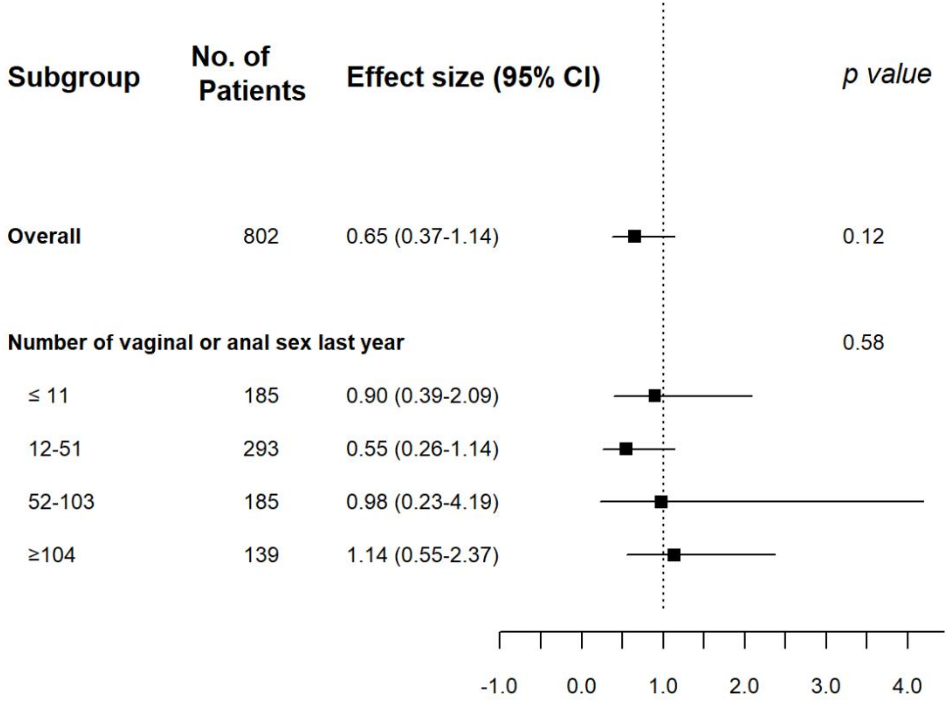

(A) Any HPV infection

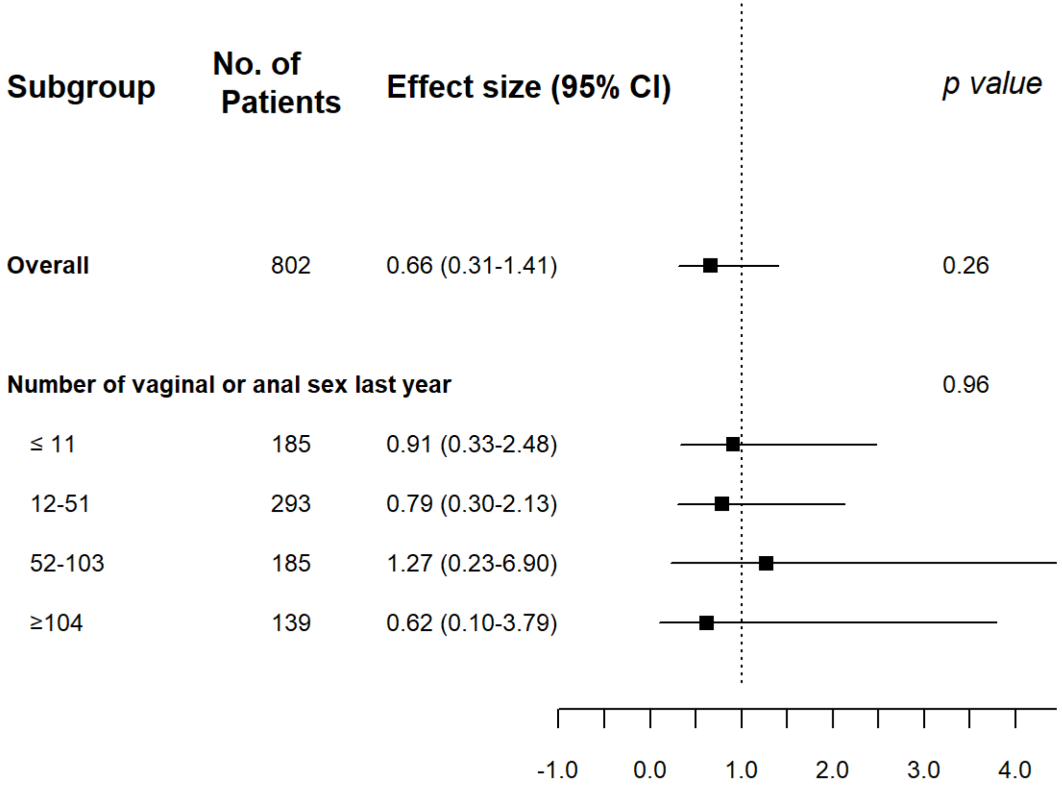

(B) High risk HPV infection
